# Physical mapping of repetitive DNA suggests 2n reduction in Amazon turtles *Podocnemis* (Testudines: Podocnemididae)

**DOI:** 10.1371/journal.pone.0197536

**Published:** 2018-05-29

**Authors:** Manoella Gemaque Cavalcante, Carlos Eduardo Matos Carvalho Bastos, Cleusa Yoshiko Nagamachi, Julio Cesar Pieczarka, Marcelo Ricardo Vicari, Renata Coelho Rodrigues Noronha

**Affiliations:** 1 Centro de Estudos Avançados da Biodiversidade, Laboratório de Citogenética, Instituto de Ciências Biológicas, Universidade Federal do Pará, Belém, Pará, Brasil; 2 Departamento de Biologia Estrutural, Molecular e Genética, Universidade Estadual de Ponta Grossa, Ponta Grossa, Paraná, Brasil; Universita degli Studi di Roma La Sapienza, ITALY

## Abstract

Cytogenetic studies show that there is great karyotypic diversity in order Testudines (2n = 26–68), and that this may be mainly attributed to the presence/absence of microchromosomes. Members of the Podocnemididae family have the smallest diploid numbers of this order (2n = 26–28), which may be a derived condition of the group. Diverse studies suggest that repetitive-DNA-rich sites generally act as hotspots for double-strand breaks and chromosomal reorganization. In this context, we used fluorescent *in situ* hybridization (FISH) to map telomeric sequences (TTAGGG)n, 45S rDNA, and the genes encoding histones H1 and H3 in two species of genus *Podocnemis*. We also observed conservation of the 45S rDNA and H1 histone sequences (probable case of conserved synteny), but multiple conserved and non-conserved clusters of H3 genes, which colocalized with the interstitial telomeric sequences in the *Podocnemis* genome. Our results suggest that fusions have occurred between macro and microchromosomes or between microchromosomes, leading to the observed reduction in diploid number in the family Podocnemididae.

## Introduction

The members of order Testudines may be subdivided in two suborders (Cryptodira and Pleurodira) and comprise one of the oldest lineages of existing vertebrates [1]. Studies have revealed a high degree of karyotypic variation in this order; the diploid numbers (2n) range from 26 in *Peltocephalus dumerilianus* (Pleurodira, Podocnemididae) [2,3] to 68 in *Carettochelys insculpta* (Cryptodira, Carettochelyidae) [4,5], with 2n = 52 reported as the most frequent diploid number [5]. The karyotypic diversity of the Testudines is attributed mainly to the presence/absence of microchromosomes. In suborder Cryptodira, the 2n ranges from 48 to 68 and numerous microchromosomes are seen [4–6]. In suborder Pleurodira, representatives of Chelidae have high diploid numbers and observable microchromosomes, with the 2n ranging from 50 to 58 [5–7]. Species of superfamily Pelomedusoidea have the smallest diploid numbers: the 2n ranges from 34 to 36 in Pelomedusidae, which have a few microchromosomes [7], and from 26 to 28 in Podocnemididae, which lack microchromosomes [3,8–10]. Cytogenetic studies of the Podocnemididae (*Erymnochelys*, *Peltocephalus* and *Podocnemis*) have suggested that their smaller diploid numbers represent a derived condition (chromosomal reduction) that was likely caused by multiple chromosomal rearrangements [5,7,9,11].

Chromosome mapping of telomeric sequences has been widely used to identify chromosomal rearrangements between the karyotypes of different vertebrate lineages, including various mammals [12–15] amphibians [16,17] and fishes [18,19]. In diverse organisms, the presence of interstitial telomeric sequences, often in association with heterochromatic regions, appear to represent remnants of chromosomal rearrangements that have contributed to reorganizing the genomic architecture and providing new chromosomal forms during evolution [17,20–24]. In chelonians, interstitial telomeric sequences have been identified and examined in *Podocnemis unifilis*; the authors of these studies proposed that the interstitial telomeric sequences were due to the amplification of telomere-like sequences [10] or represented remnants of chromosomal fusions that reduced the diploid number [5,10].

The grouped organization of rDNA and histone genes makes these sequences useful as chromosomal markers for the study of chromosomal variation and genomic organization in many groups of eukaryotes [25]. High mutation rates in intergenic regions of multigenic families represent an important source of genetic variability and can generate sites that are prone to undergoing double-strand breaks (DSB), which also promotes chromosomal reorganization during karyotypic evolution [19,22,26,27]. In family Podocnemididae, studies suggest that the 45S rDNA located on the first chromosome pair is conserved [3,5,10]. Histone genes have been mapped in diverse organisms [25,28–31], but physical chromosome mapping of histone genes had not previously been reported in any member of order Testudines.

It has been suggested that sites rich in repetitive DNA act as hotspots for DSB and chromosomal reorganization [19,32–34]. This proposal has been supported by data from the *in situ* mapping of multigenic families, microsatellite expansions and transposable elements in the regions of syntenic breaks, as well as by studies of the chromosomal organizations of many groups [19,32–36]. Because repetitive-DNA-rich regions contain many paralogous genes copies, they facilitate DSB, non-homologous recombination and Robertsonian fusion-based rearrangements [19,33,37]. These regions also undergo sequence exchanges and duplications of subtelomeric regions, such as expansions of multigenic families located near telomeres [38].

The fusion of microchromosomes between themselves and/or with macrochromosomes is considered to be the main mechanism of diploid number reduction in amniotes and tetrapods [39]. In scaled reptiles, it is believed that the large numbers of microchromosomes predicted as the ancestral state were reduced by such fusions [40–42]. In Testudines, some cytogenetic data strengthen the chromosome evolution hypothesis of the group, as ribosomal DNA and nucleolus organizer region, localized in microchromosomes in testudinatas with high diploid number (2n = 50–58) [5,43,44], while for the family Podocnemididae the same markers are reported located on the first chromosome pair [3,5,10].

Here, in an effort to improve our understanding of the chromosomal evolution and genomic dynamics of *Podocnemis* (Pleurodira, Podocnemidae), we used fluorescent *in situ* hybridization (FISH) to probe the telomeric, 45S rDNA and histone H1 and H3 sequences in two species of the genus (*Podocnemis expansa* and *Podocnemis unifilis*).

## Materials and methods

### Specimens and approval

We studied two species of genus *Podocnemis*, *Podocnemis expansa* and *Podocnemis unifilis*, utilizing specimens kept in the Zoobotanical Park Adhemar Monteiro, Capitão Poço, Pará, Brazil. This study was conducted in strict accordance with the ethical recommendations for the use and management of chelonians in research, under a protocol approved by Ethics Committee on Experimental Animal Research (license number 68–2015) and Biodiversity Information and Authorization System (SISBIO; license number 42642–5).

### Chromosomal preparation, DNA extraction and probe production

Lymphocyte culture and chromosomal preparation were performed as described by Viana et al. [45]. Genomic DNA was purified from the muscle tissues and blood specimens using the conventional proteinase K and phenol/chloroform extraction method [46]. The obtained DNA was diluted in elution buffer and kept at– 20°C until use. The genes encoding histones H1 and H3 were polymerase chain reaction (PCR) amplified using the following primers: 5'-AGA RGA GCG GCG TGT-3' and 5’-CYT CTT CRC CTT CYT KG-3’ for histone H1; and 5′-ATG GCT CGT ACC AAG CAG AC(ACG) GC-3′ and 5′-ATA TCC TT(AG) GGC AT(AG) AT(AG) GTG AC-3′ for histone H3, both designed by Cabral-de-Mello et al. [47]. The amplification reaction set up: genomic DNA = 80 ng, forward primer = 0.2 μM, reverse primer = 0.2 μM, dNTPs = 0.16 mM, Taq DNA Polymerase (Invitrogen) = 1 U, MgCl2 = 1.5 mM, reaction buffer 1× (200 mM Tris, pH 8.4, 500 mM KCL). The amplification program set up: 4min– 95°C/(1min—95°C / 1min—60°C / 2min—74°C) 30 cycles / 5min—74°C. The general telomeric sequence of vertebrates (TTAGGG)n was obtained as described by Ijdo et al. [48]. To construct the 45S rDNA probe, we used the pTa71 plasmid, which contains the 5.8S, 18S and 28S genes and their respective intergenic spacers from *Triticum aestivum* [49]. The probes were nick-translation-labeled with biotin 14-dATP or digoxigenin 16-dUPT using the BioNick Labeling System (Invitrogen) and a DIG-Nick kit (Roche Applied Science), respectively.

### Fluorescence *in situ* hybridization (FISH)

FISH was performed as described by Pinkel et al. [50], with some modification. The signals were detected with avidin-CY3 (Sigma) and antidigoxigenin-FITC (Roche). Chromosomes were counterstained with 4´,6-diamidino-2-phenylindole (DAPI; 0.2 μg mL^-1^) in Vectashield H-100 mounting medium (Vector) and analyzed under an epifluorescence microscope (Nikon H550S). The chromosomes were organized by size and categorized as metacentric (m), submetacentric (sm), subtelocentric (st) or acrocentric (a) as previously described [51]. Approximately 30 metaphase spreads of each species were analyzed to determine the diploid number, karyotypic formula and the presence/absence of interstitial telomeric sequences, rDNA and histones H1 or H3.

## Results

**Fig 1:**
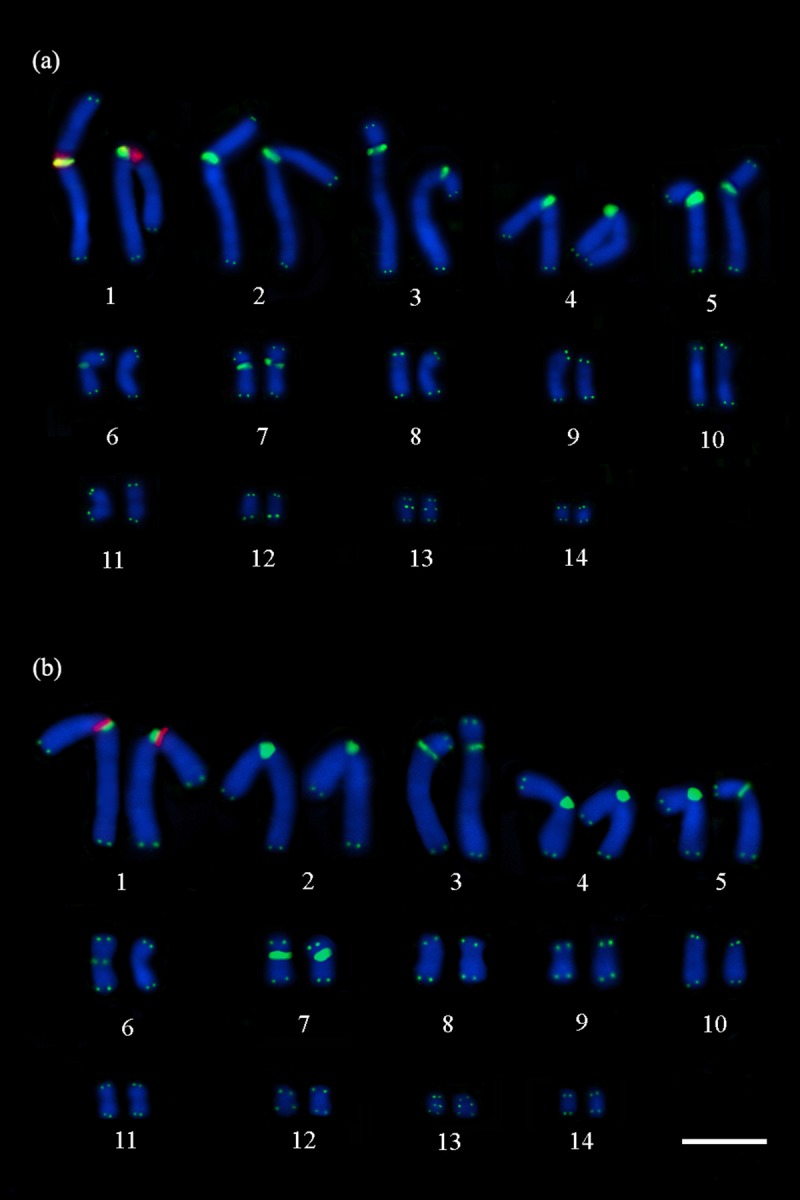
Double FISH with telomeric (TTAGGG)n probes and 45S rDNA. Telomeric (TTAGGG)n probes (in green) show the interstitial telomeric sequences in the pairs 1–5, 7 and 13, and in a single chromosome of the pair 6, and 45S rDNA (in red) in the proximal region of the short arm of the pair 1, adjacent to the interstitial telomeric sequences in (a) *P*. *expansa* and (b) *P*. *unifilis*. Scale bar = 10μm.

**Fig 2:**
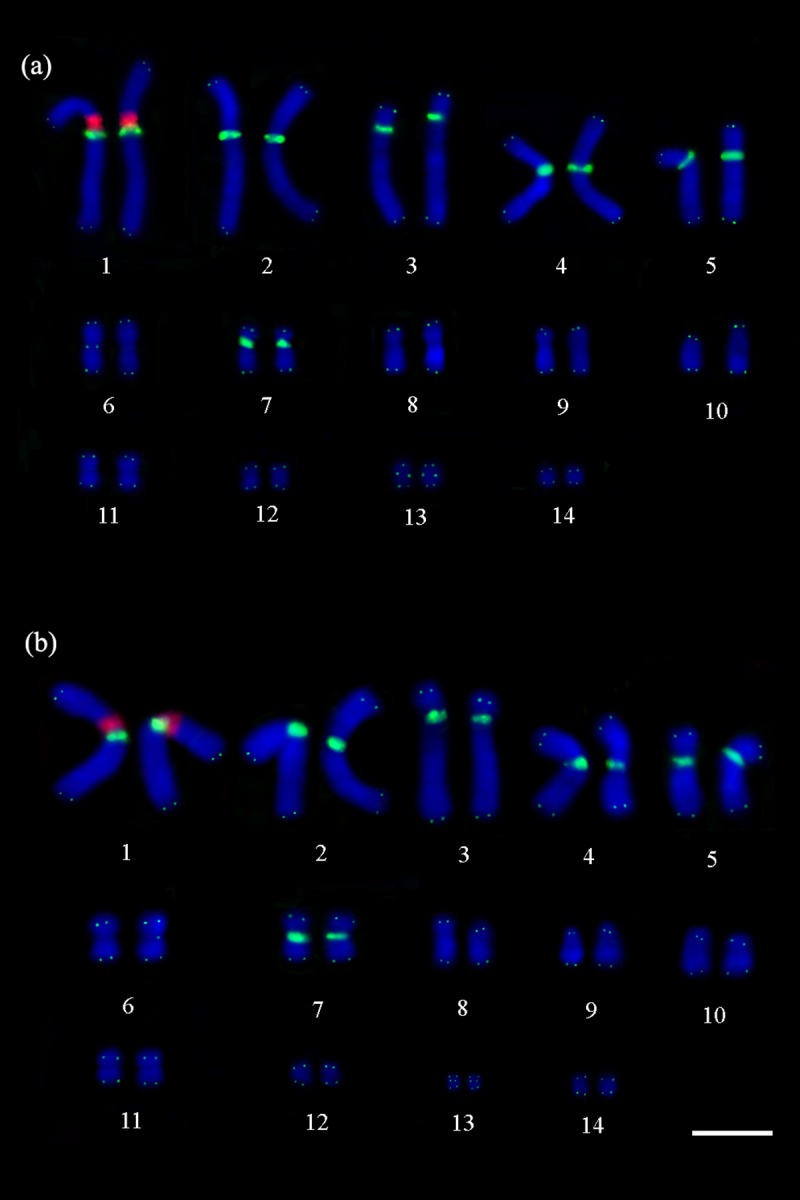
Double FISH with telomeric (TTAGGG)n probes and H1 histone genes. Telomeric (TTAGGG)n probes (in green) show the interstitial telomeric sequences in the pairs 1–5, 7 and 13, and in a single chromosome of the pair 6, and H1 histone genes (in red) in the proximal region of the short arm of the pair 1, adjacent to the interstitial telomeric sequences in (a) *P*. *expansa* and (b) *P*. *unifilis*. Scale bar = 10μm.

**Fig 3:**
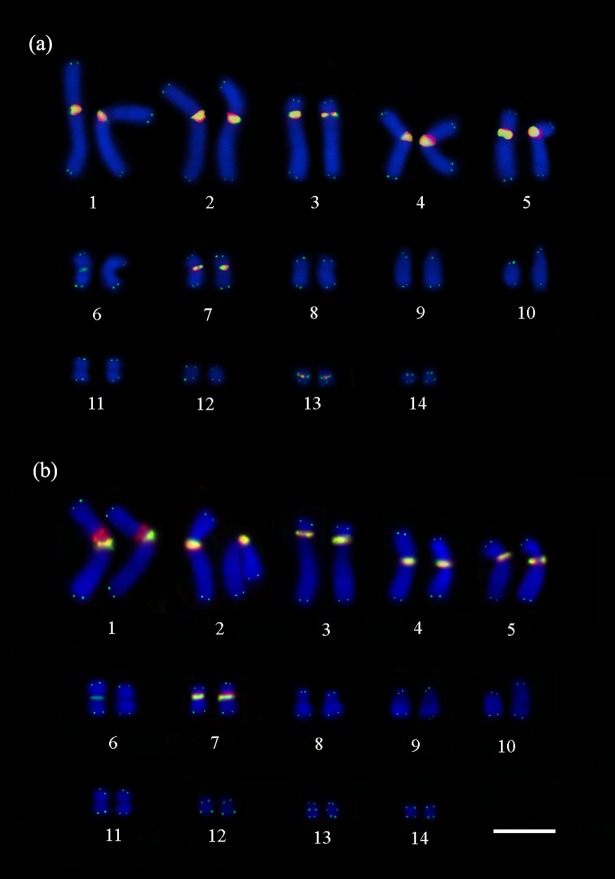
Double FISH with telomeric (TTAGGG)n probes and H3 histone genes. Telomeric (TTAGGG)n probes (in green) show the interstitial telomeric sequences in the pairs 1–5, 7 and 13, and in a single chromosome of the pair 6, and H3 histone clusters (in red) co-localized with interstitial telomeric sequences and distributed in the pericentromeric regions of the pairs 1–5, 7 and 13 in (a) *P*. *expansa* and the pairs 1–5 and 7 in (b) *P*. *unifilis*. Scale bar = 10μm.

The two species presented a diploid number of 28 chromosomes. *P*. *expansa* had a fundamental number (FN) of 54 and a karyotypic formula of 24m/sm + 2st + 2a, while *P*. *unifilis* had FN = 52 and a karyotypic formula of 22m/sm + 2st + 4a (Figs 1–3). The main karyotypic difference between the two species was in chromosome pair 9, which was submetacentric in *P*. *expansa* and acrocentric in *P*. *unifilis*. Both species also showed a size heteromorphism for pair 10. No heteromorphic sex chromosome was found in either species.

Telomeric (TTAGGG)n signals were observed in the distal regions of all chromosome pairs in *P*. *expansa* and *P*. *unifilis*. In addition, interstitial telomeric sequences signals were detected on pairs 1–5, 7 and 13 in both species; besides those signals, it was detected interstitial telomeric sequences in a chromosome of pair 6, in a single homologue, in the two species (Figs 1–3).

The 45S rDNA sites were found in the proximal region of the short arm of submetacentric pair 1 in both species. Double-FISH showed that the 45S rDNA and the interstitial telomeric sequences signals in the first chromosome pair are adjacent in *P*. *expansa* and *P*. *unifilis* (Fig 1).

Similar to the results obtained from FISH with rDNA, the H1 histone sequence was localized in the proximal region of the short arm of the first chromosome pair in both species. Double-FISH revealed that the signals of the histone H1-encoding genes are adjacent with the interstitial telomeric sequences in both species (Fig 2).

*In situ* localization of the histone H3-encoding sequences revealed that clustered in the pericentromeric regions of six chromosome pairs (pairs 1–5 and 7) in *P*. *unifilis* (Fig 3B). Similar signals were observed in *P*. *expansa* specimens, and also revealing clusters of histone H3-encoding sequences in an additional chromosome pair (pairs 1–5, 7 and 13) (Fig 3A). Double-FISH showed that the histone H3 signals consistently colocalized with the interstitial telomeric sequences of both species, except for the interstitial telomeric sequences present in the single chromosome from pair 6 in both species and in pair 13 of *P*. *unifilis* (Fig 3).

## Discussion

Podocnemididae have the smallest diploid numbers of the order Testudines, with 2n ranging from 26 to 28 chromosomes [3,10]. Our results corroborate those previously obtained for *P*. *expansa* and *P*. *unifilis*, in that we observed 2n = 28 chromosomes, with no microchromosomes [7–11,52]. We also observed evidence of possible chromosome fusions in these species. Our data support the hypothesis that the diploid number has undergone reduction in Podocnemididae and suggest a few chromosomal sites that may have been involved in these genomic reorganization events.

Our cytogenetic data revealed the presence of size heteromorphism for pair 10 in the karyotypes of *P*. *expansa* and *P*. *unifilis*, which is consistent with the data obtained by Noronha et al. [10] for *P*. *unifilis*. The authors of the prior paper suggested that this might reflect a size variation in the constitutive heterochromatin of one of the homologous chromosomes, which would have originated through uneven crossover(s), transposition(s), and/or duplication(s) in *cis*. However, whereas Noronha et al. [10] did not observe heteromorphism of chromosome pair 10 in *P*. *expansa*, we observed such heteromorphism in the present study. This apparent discrepancy can be explained by the shortening of the chromosomes that occurs during the chromosomal preparation method used in the previous paper, complicating the identification of heteromorphism. Our analysis further showed that this karyotypic variation did not involve the 45S rDNA, (TTAGGG)n or histone H1 and H3 sequences.

Our identification of interstitial telomeric sequences sites in the pericentromeric regions of both species corroborates the findings of Montiel et al. [5] and Noronha et al. [10] for specimens of *P*. *unifilis*, but contrasts with the lack of such sites reported by Noronha et al. [10] for *P*. *expansa*. We speculate that the interstitial telomeric sequences of the previously studied examples of *P*. *expansa* could have undergone successive losses and/or degenerations, leading to a gradual shortening of non-functional telomeric matrices [53]. In this context, such interstitial telomeric sequences would be very short and might not be detected by the techniques previously used for their visualization [54,55]. The shortening of the non-functional telomeric matrix could be a possible cause for the visualization of the interstitial telomeric sequences in a single homologue of the chromosome pair 6 in the present study. In addition, not all chromosomal fusions retain telomeric DNA repeats at the fusion points. The lack of telomeric hybridization signals at putative fusion sites may therefore suggest that the chromosome breakage that preceded the fusion event occurred within the chromatin proximal to the telomeric region [54].

Our preliminary analysis indicated that the interstitial telomeric sequences in the pericentromeric chromosomal regions of *P*. *expansa* and *P*. *unifilis* can be can be categorized as heterochromatic interstitial telomeric sequences. This suggests that these regions may have been involved in the diploid number reduction of Podocnemididae, since they are considered to be unstable regions where chromosomal rearrangements may occur [20,56,57]. The fusion of microchromosomes between themselves and/or with macrochromosomes is considered to be the main mechanism of diploid number reduction in amniotes and tetrapods [39]. In scaled reptiles, it is believed that the large numbers of microchromosomes predicted as the ancestral state were reduced by such fusions [40–42]. In lizards, few microchromosomes are found, and some chromosomal pairs are composed of tandem-fused chromosome segments that have homologies with microchromosomes; this suggests that the karyotypes of lizards probably arose via the in-tandem fusion of microchromosomes [58]. In this context, our detection of interstitial telomeric sequences in the pericentromeric region of seven chromosome pairs of *P*. *expansa* and *P*. *unifilis* reinforce the hypothesis that these interstitial telomeric sequences represent telomeric DNA remnants at points where micro- and macrochromosomes, or in tandem between microchromosomes, underwent fusion during evolution. However, it is important to emphasize that the interstitial repetitions of TTAGGG observed in this manuscript may also represent effect of telomeric sequence amplification, or like-telomeres regions, because generally these repetitions are lost, as previously reported in specimens of *P*. *expansa* [10].

**Fig 4:**
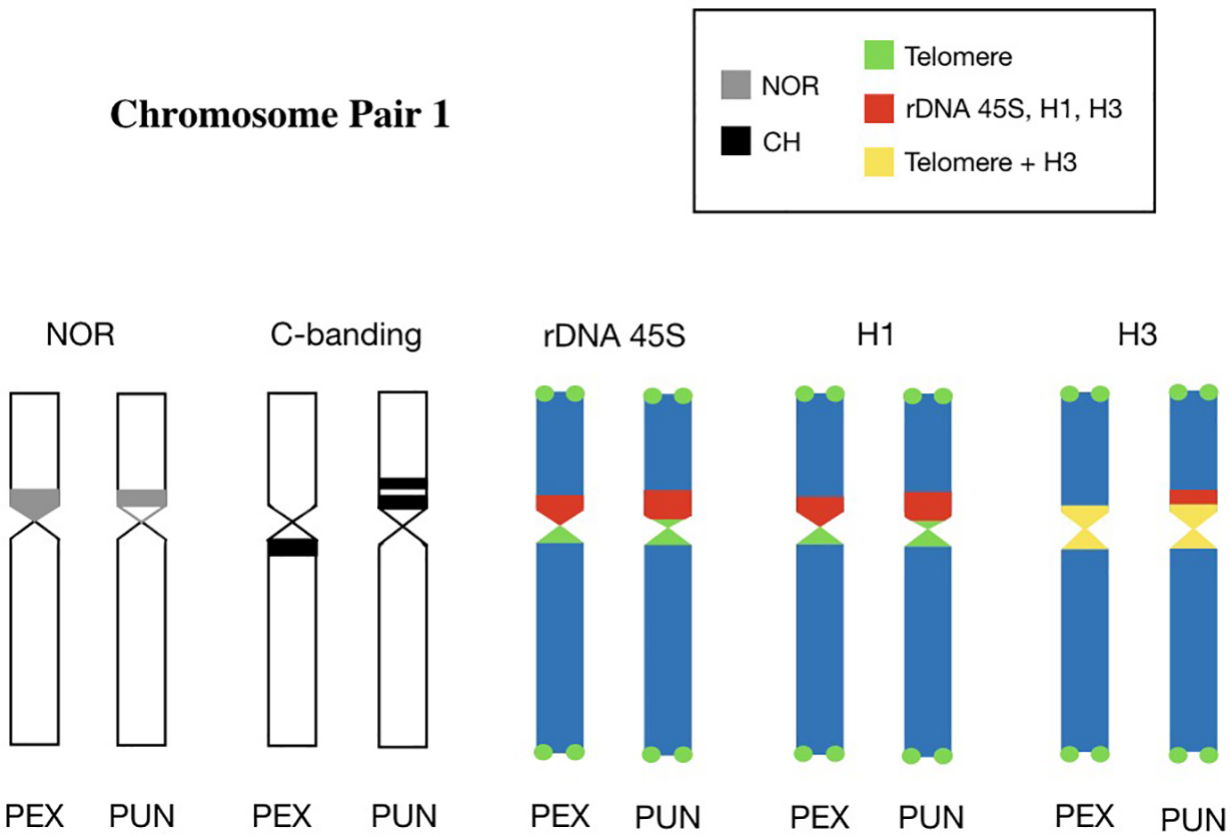
Ideogram of the common chromosomal region of the first pair of *Podocnemis expansa* and *Podocnemis unifilis*. Representation of the relationships of the repetitive sequence investigated in this manuscript (telomeric sequences in green; 45S rDNA and histones H1 and H3 in red) in respect with Nucleolus Organizer Regions (NOR) and C-bands described in already published paper [5,10], indicating a probable segment conserved synteny for the family Podocnemididae. The acronyms “PEX” make reference to species *P*. *expansa* and “PUN” to *P*. *unifilis*.

Studies have demonstrated that the 45S rDNA is localized in chromosome pair 1 of Podocnemididae [3,5,10]. In *P*. *expansa* and *P*. *unifilis* the nucleolus organizer region (NOR) is flanked by regions that display CMA3 signals, indicating that the 45S rDNA region of the first chromosomal pair in these species is rich in GC base pairs [10]. In this context, we propose that the first chromosome pair can be considered as a marker, with synapomorphic characteristic to the Podocnemididae family. Or yet, it is possible that genes preserved between representatives of the Podocnemididae family to signal a case of conserved synteny, because some genes tend to stay together throughout evolution and remain as conserved syntenyc blocks in a wide range of species [59–62]. So we constructed the ideogram that represent the physical chromosome mapping indicating a probable conserved synteny segment for the family Podocnemididae (Fig 4).

Previous studies found that 45S rDNA sites were localized in microchromosomes of the following: *Hydromedusa tectifera* (Pleurodira, Chelidae), which has 2n = 58 [43]; members of genus *Trachemys* (Cryptodira, Emydidae), which has 2n = 50 [44]; *Sternotherus odoratus* (Cryptodira, Kinosternidae), which has 2n = 56; *Emydura macquarii* (Pleurodira, Chelidae), which has 2n = 50; and *Chelodina oblonga* (Pleurodira, Chelidae), which has 2n = 54 [5]. The microchromosome localizations of 45S rDNA sequences in species with higher diploid numbers strongly support the idea that chromosomal fusions took place between rDNA-carrying microchromosomes and macrochromosomes during the evolution of chromosome pair 1 of Podocnemididae.

Although the physical mapping of histone genes have been done in some organisms, as invertebrates [25,28,31] and fishes [29,30], the present work is the first to report the *in situ* location of histone gene sequences in members of order Testudines. We found genes encoding histones H1 and H3 in the proximal region of the short arm of the first chromosome pair, indicating that this site is likely to be the main histone cluster for *P*. *expansa* and *P*. *unifilis*. This reinforces the notion that this region houses several repetitive sequences and represents a synapomorphic characteristic of family Podocnemididae, or yet a case of conserved synteny. However, the most striking case was the location of histone H3. Although the histone genes are very conserved within species, the organization of their clusters within the genome may be heterogeneous [28,30,63]. The difference in the distribution pattern of many H3 sites not correlated with H1 sites suggests an evolutionary dichotomy between those sequences in genome of *P*. *expansa* and *P*. *unifilis*. Some studies have suggested that the H3 sequences may be dispersed throughout genomes by ectopic recombination, invasion of transposable elements (TE), and/or circular DNA [30,31]. In fishes, Pucci et al. [64] demonstrated that parts of TE may be found in the intergenic regions of histone sequences, and suggested that such elements could help disperse copies of histone genes throughout a genome. Thus, it is likely that the dispersion of histone H3 in the studied species may be associated with TE insertions and/or genetic hitchhiking.

Non-reciprocal sequence exchanges and duplications of subtelomeric regions are frequent, especially when there is expansion of multigenic families close to telomeres [38]. Histone sequences have features that are common to chromosome breakage regions, in that they are arranged in tandem repeats, localized at pericentromeric or subtelomeric chromosome regions, display transposition ability when invaded by TE, and exhibit high intra- and inter-chromosomal recombination rates. In a similar pathway, interstitial telomeric sequences are associated with hotspots for chromosomal breakage and are involved in DSB repair; they appear to represent a favorable substrate for chromosome breakage and may thus promote genomic instability (for details, see [55]). In the present study, the colocalization of H3 histone with interstitial telomeric sequences in pericentromeric regions of the two species also suggests that non-homologous recombination may have acted in the dispersion of these sequences. Such sequences would logically trigger chromosomal rearrangements [20], since interstitial telomeric sequences create chromosomal instability and are prone to DSB [65,66]. This would support mainly end-to-end fusions, which could cause the observed reduction to 2n = 28.

## Conclusions

In conclusion, we herein report that the karyotypes of two representative members of *Podocnemis* lack microchromosomes but harbor interstitial telomeric sequences. We provide evidence that the fusions of macro- and microchromosomes or in tandem between microchromosomes have occurred during the chromosomal evolution of this group, reducing the diploid number (2n = 28). Furthermore, the genomic locations of rDNA and genes encoding histone H1 are conserved on the first chromosome pair of *Podocnemis*, may represent conserved syntenyc blocks, whereas the genes encoding histone H3 are distributed in multiple conserved and non-conserved clusters that colocalized with interstitial telomeric sequences, can indicate non-homologous recombination or associated with TEs insertions and genetic hitchhiking.
